# Morphology Comparison of Different Mineral Coarse Aggregates Produced from Two Typical Crushers

**DOI:** 10.3390/ma19142972

**Published:** 2026-07-10

**Authors:** Saisai Zhang, Shan Li, Ziyu Zhao, Zhengwei Yang

**Affiliations:** National Key Laboratory of Solid Rocket Propulsion, Rocket Force University of Engineering, Xi’an 710025, China; lishan8882026@163.com (S.L.); ziyuzhao625@163.com (Z.Z.); yangzhengwei1136@163.com (Z.Y.)

**Keywords:** coarse aggregates, morphological characteristics, crushing operations

## Abstract

Morphological characteristics of coarse aggregates, namely shape, angularity and surface texture, are closely related to rock mineral compositions and crushing mechanisms. Aggregates with different mineral compositions should be crushed using a suitable crusher. To explore the influence of mineral and crusher types on aggregate morphology, this study investigated the effect of the mineral compositions and crushing operations on the morphologies of coarse aggregates. Six types of major rock-forming minerals (i.e., quartz, amphibole, potassium feldspar, sodium feldspar, calcite and pyroxene) were selected and two typical crushers (i.e., jaw crusher and impact crusher) were used. The morphology parameters (i.e., angularity, texture, sphericity and F&E) of coarse aggregates were measured using the Aggregate Image Measurement SystemII (AIMSII). Further, the morphologies of different aggregates produced by two crushers were compared. The results showed that the angularity values of some aggregates crushed by a jaw crusher (average 3451) were bigger than those by an impact crusher (average 3067) and the angularity of the harder mineral was less affected by the crusher. For surface texture, there was no significant difference between these two crushers, with average texture index of 333.83 for the impact crusher and 332.67 for the jaw crusher, indicating that the surface texture of aggregates was mainly affected by their compositions and barely influenced by the crushing operations. The shape results indicated that the impact crusher produced more cubical aggregate particles compared to the jaw crusher, with average sphericity of 0.666 versus 0.607, whereas the jaw crusher produced aggregates with more elongated or flat particles, with an average F and E index of 3.331 versus 2.726. This study fills the research gap that few previous investigations focused on—the crushing morphology of single mineral aggregates—and the findings help us to understand the effect of the mineral compositions and crushing operations on the morphologies of coarse aggregates, which in turn guides the selection of suitable crushers for different minerals.

## 1. Introduction

Asphalt mixture is a multi-phase composite material composed of asphalt, fillers, and coarse and fine aggregates, in which mineral aggregates account for about 90% of its mass. The properties of mineral aggregates greatly affect the performance of asphalt mixtures. Various regulations on the characteristics of aggregates have been made in different regions [1,2]. The Chinese Technical Specification for Construction of Highway Asphalt Pavements puts forward requirements for the physical properties of aggregates (i.e., apparent relative density and water absorption), mechanical properties (i.e., crushing value, Los Angeles abrasion loss and firmness value), shape properties (i.e., percentage of flat–elongated particles, particle size and the number of crushed surfaces) and the adhesion to asphalt [3]. The Strategic Highway Research Program (SHRP) stipulates the mechanical properties of aggregates (i.e., stability and toughness), composition properties (i.e., harmful substances and clay content) and shape properties (i.e., angularity and percentage of flat–elongated particles) [4]. The British Standard makes regulations on the physical properties of aggregates (i.e., density, water absorption), mechanical properties (i.e., crushing resistance, abrasion resistance, thermal shock resistance and firmness), composition properties (i.e., chemical composition and content of coarse and light pollutants), shape properties (i.e., percentage of flat particles and the number of crushed surfaces) and the adhesion to asphalt [5]. Obviously, the regulations on aggregate properties mainly focus on their physical and mechanical properties, while the requirements of their morphological properties are limited.

Along with the physical and mechanical properties of aggregates, the morphological characteristics of aggregates (i.e., shape, angularity and surface texture) have a significant effect on the functional and structural performance of asphalt pavement [6,7,8]. The aggregates with cubic shape, multi-angular corners and rough surface have a positive effect on the mechanical properties of the asphalt mixture [9,10]. Aggregate shape is the profile boundary of the particle [11]. The cubical aggregates have good internal cohesion and are able to form a stable aggregate skeleton, which is beneficial to the anti-fatigue performance of the mixture [12]. Aggregate angularity is the corner sharpness of the particle [13]. It can provide the internal friction between aggregates and improve the rut resistance of asphalt mixtures significantly [14,15]. Aggregate texture is the surface roughness of the aggregate [16]. Rough surface texture can improve the bond strength between aggregates and asphalt to achieve higher resistance to moisture damage [17,18]. It can be seen that there is a strong correlation between the morphological characteristics of aggregates and the road performance of mixtures [19].

The morphology of the aggregate is primarily a function of the parent rock type and crushing process [20]. Due to the different mineral compositions of various parent rocks, there are great differences in their hardness and structure [21]. In order to control the morphology of the aggregate, different rocks should be produced by their suitable crushing processes. The Iowa Department of Transportation (DOT) investigated the effect of cone and horizontal shaft impactor on different aggregates, and found that aggregates produced from the horizontal shaft impactor had a higher void in mineral aggregate (VMA) compared to cone-crushed aggregates [22]. Huber analyzed the shape of limestone aggregates crushed by cone and vertical shaft impactors, respectively, and the results showed that there were more flat or elongated particles in the cone-crushed aggregates compared to aggregates produced from vertical shaft impactor [23]. So far, limited studies focus on investigating the effect of crushing mechanism on the morphological properties of aggregates to improve the aggregate morphology fundamentally. Most existing research only considers the single mineral hardness index and rarely systematically explores the coupling interaction between multiple mineral inherent characteristics and different crushing modes, which is the key research gap addressed in this paper [24].

To quantitatively reveal the independent influences of parent rock mineralogy and crushing mechanism on aggregate morphological characteristics, this paper carries out a systematic comparative test. The six most common rock-forming minerals, including quartz, amphibole, potash feldspar, sodium feldspar, calcite and pyroxene, were selected. Two typical crushing mechanisms were adopted, including the extrusion crushing mechanism represented by the jaw crusher and the impact crushing mechanism represented by the impact crusher. Minerals with different hardness or parent rock properties will form completely different angularity, surface texture and shape characteristics under extrusion crushing and impact crushing. The morphologies (i.e., angularity, texture, sphericity and F&E) of aggregates crushed by two different crushers were analyzed by the Aggregate Image Measurement SystemII (AIMSII). Through comparing the morphology characteristics, the most dominant factor in affecting aggregate morphologies can be determined, which is useful to choose the suitable crushing equipment for different types of aggregates. The test results are expected to clarify the differentiated particle morphology evolution law of single mineral aggregates under two crushing modes, which fills the research gap that few existing studies focus on: the morphological variation rule of individual single-mineral aggregates under different crushing mechanisms.

## 2. Experiment Descriptions

**Figure 1:**
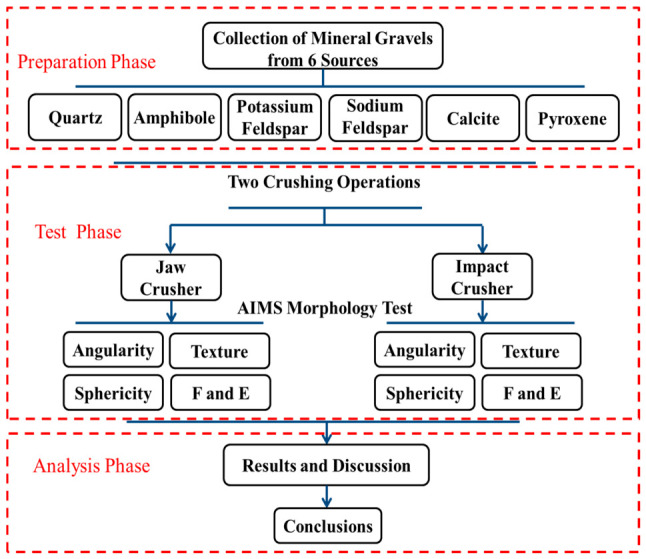
Experimental program.

In this study, six kinds of mineral gravels were selected from six different cities in China. The jaw crusher and impact crusher were used to crush these minerals, respectively. The morphologies of these mineral aggregates were analyzed using AIMS. The schematic of the experimental program adopted in this paper is shown in Figure 1.

### 2.1. Materials

In this study, six major rock-forming minerals (quartz, potassium feldspar, sodium feldspar, calcite, pyroxene and amphibole) were selected, which are the basic composition of commonly used aggregates. The six selected minerals are widely distributed in natural rocks and cover a full range of hardness, which can accurately represent typical single-mineral aggregates for this comparative test. The sources and properties of these minerals are shown in Table 1.

**Figure 2:**
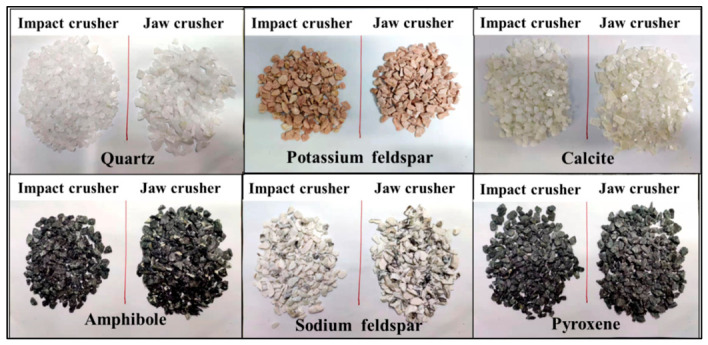
Coarse aggregates, crushed by two types of crushing equipment.

These six mineral gravels were crushed by a jaw crusher and impact crusher, respectively. After crushing, all aggregates were screened to obtain single-sized particles with a size range of 4.75 mm to 9.5 mm. The screened aggregates were washed and dried, and the finished samples were presented in Figure 2.

### 2.2. Crushing Operations

#### 2.2.1. Jaw Crusher

**Figure 3:**
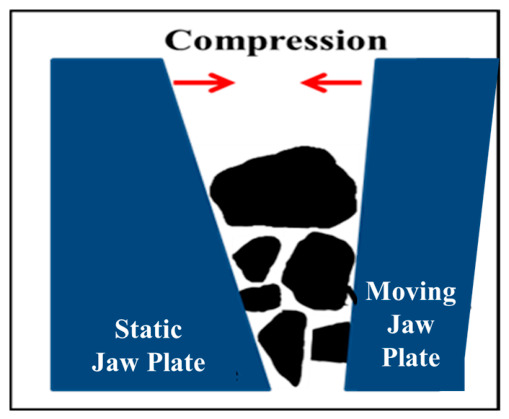
Schematic diagram of jaw crusher.

In the jaw crusher, the breakage is primarily based on the compression action. It is mainly composed of a moving jaw plate and a static jaw plate, which form a certain angle with each other. The schematic is shown in Figure 3. The reciprocating movements of the moving jaw plate can produce sufficient compression for aggregates to make them break. For the laboratory jaw crusher adopted in this experiment, the maximum feed size was 125 mm, and the discharge opening was fixed at 8 mm throughout all crushing tests. This equipment can crush minerals with Moh’s hardness up to eight, and its working efficiency ranges from 45 to 55 kg per hour.

#### 2.2.2. Impact Crusher

**Figure 4:**
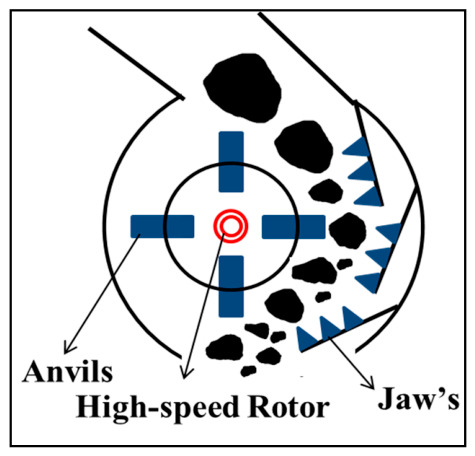
Schematic diagram of impact crusher.

The impact crusher mainly works on the impact mechanism. It consists of a horizontal shaft attached to a high-speed rotor mounted with fixed anvils, as shown in Figure 4. First, the rocks collide with the rapidly rotating anvils. Further, the crushed aggregates collide with the jaws fixed in a crushing chamber. This two-step collision generates high impact action on the aggregates and results in the breakage of particles in a short time. For the laboratory impact crusher used in this study, the maximum feed size was 80 mm, and the discharge opening was uniformly set to 8 mm in all crushing procedures. This equipment can process materials with Moh’s hardness no higher than 7.5, with a working efficiency of 50–60 kg per hour.

### 2.3. Aggregate Image Measurement System

The AIMS is based on the digital image technique to measure the morphology parameters for aggregates. Research has shown that the AIMS can eliminate subjectivity by removing human influence and improving test precision and productivity. The comparison of the test accuracy and repeatability results of AIMSII (Pine Instrument Company, Grove City, PA, USA), University of Illinois Aggregate Image Analyzer (UIUIA) (University of Illinois at Urbana-Champaign, Urbana, IL, USA) and Fourier Transform Interferometer (FTI) (Zygo, Middlefield, CT, USA) showed that AIMS could better differentiate aggregates of all kinds of colors from different origins with results with wide numerical ranges for shape, angularity and texture, respectively. So AIMSII made by American Pine Instrument Company (Grove City, PA, USA) was adopted to quantify the shape characteristics for coarse aggregates in this study.

**Figure 5:**
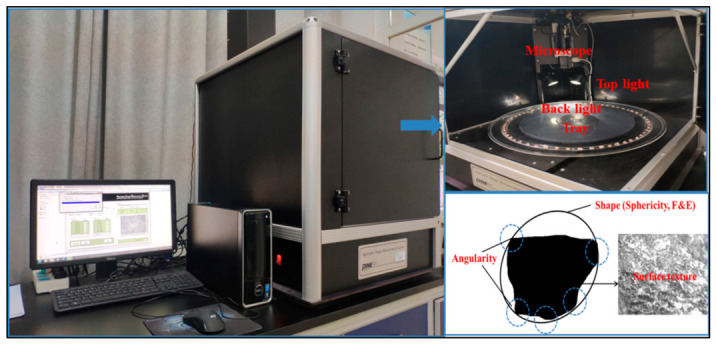
Schematic diagram of AIMS II system, adapted from [8].

AIMSII consists of a microscope camera, an aggregates tray and a back and top lighting system, which is shown in Figure 5. For coarse aggregates, three consecutive scans are conducted for analysis of the angularity, sphericity, F and E ratio, and surface texture. The first scan, namely the silhouette, is used to measure the angularity, 2D form, centroid, and aggregate dimensions along the x and y directions. The second scan measures the height of the aggregate at the centroid and the third scan measures the texture of the aggregate surface.

The morphology parameters of the coarse aggregate are shown in Table 2. Specifically, angularity represents the corner sharpness of coarse aggregates, which is defined by a gradient method. Low angularity represents the rounded particle, whereas high angularity represents the angular aggregate. Surface texture is the surface roughness of the coarse aggregate, which is determined by the gray scale images of aggregates’ surfaces. The low texture value represents the smooth surface, whereas the high texture value represents the rough surface. Sphericity is a measure of the 3D shape properties for an aggregate, which is determined by three dimensions. The higher the sphericity is, the better the shape of an aggregate is. F and E is the ratio of the longest dimension to the shortest dimension for an aggregate.

## 3. Results and Discussion

### 3.1. Angularity

**Figure 6:**
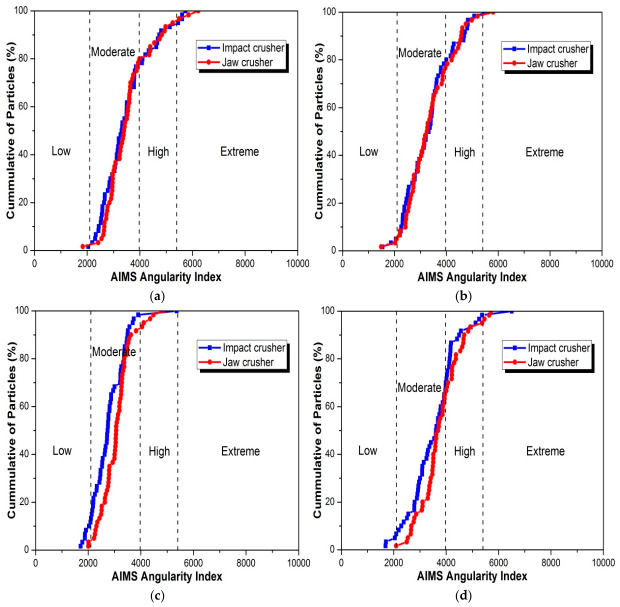

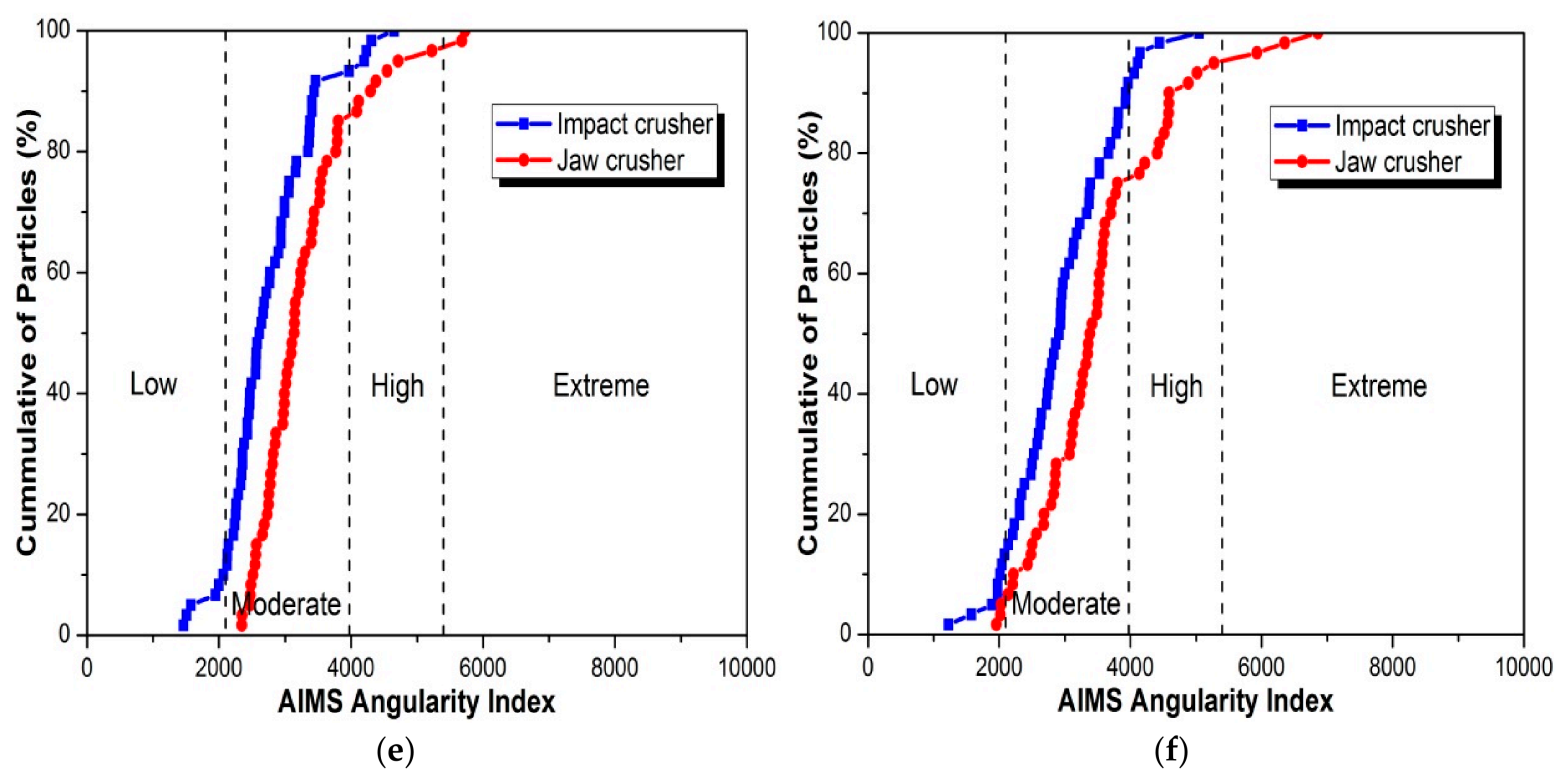
Angularity comparison of aggregates under two crushing operations. (**a**) Quartz. (**b**) Amphibole. (**c**) Potash feldspar. (**d**) Sodium feldspar. (**e**) Calcite. (**f**) Pyroxene.

**Figure 7:**
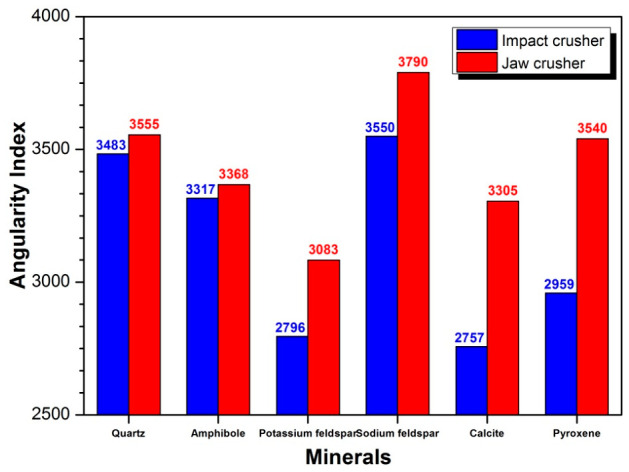
Mean angularity index of different mineral aggregates.

Angularity characterizes the jagged contour of aggregate particles, which determines the interlocking friction between aggregates. The angularity values of six mineral aggregates crushed by the jaw crusher and the impact crusher, respectively, are shown in Figure 6. It can be seen that except for quartz and amphibole, the angularity values of other aggregates crushed by the jaw crusher were bigger than that of the impact crusher. For potash feldspar and sodium feldspar, the angularity was slightly improved by the jaw crusher. For calcite and pyroxene, the aggregates crushed by the jaw crusher were obviously more angular compared to that of the impact crusher. The higher values of angularity for JC aggregates compared to IC aggregates might be because the repeated aggregate-to-hammer impact led to more blunting of aggregate sharp edges. The angularity difference between various minerals might be caused by the hardness of the mineral itself, and the angularity of the harder mineral is less affected by the crusher. The angularity differences between two crushers can be further compared according to the average values of angularity, shown in Figure 7.

The average results showed that the angularity for JC aggregates was greater, which was consistent with the above results. The 95% confidence interval of the average angularity index for jaw-crushed aggregates (3440.17) is higher than that of impact-crushed aggregates (3143.67), showing an overall higher angularity level for products from the jaw crusher. For aggregates crushed by the impact crusher, the order of the angularity index is sodium feldspar > quartz > amphibole > pyroxene > potash feldspar > calcite. For aggregates crushed by the jaw crusher, the sodium feldspar still had the largest angularity index, followed by the quartz, pyroxene, amphibole, calcite and potash feldspar. It can be seen that the jaw crusher can improve the angularity of certain aggregates, especially for calcite and pyroxene, but it can also be found that the angularity of aggregates is affected by its own composition at the same time. Therefore, for aggregates containing a lot of calcite or pyroxene, the angularity of the aggregate can be improved by a jaw crusher.

### 3.2. Surface Texture

**Figure 8:**
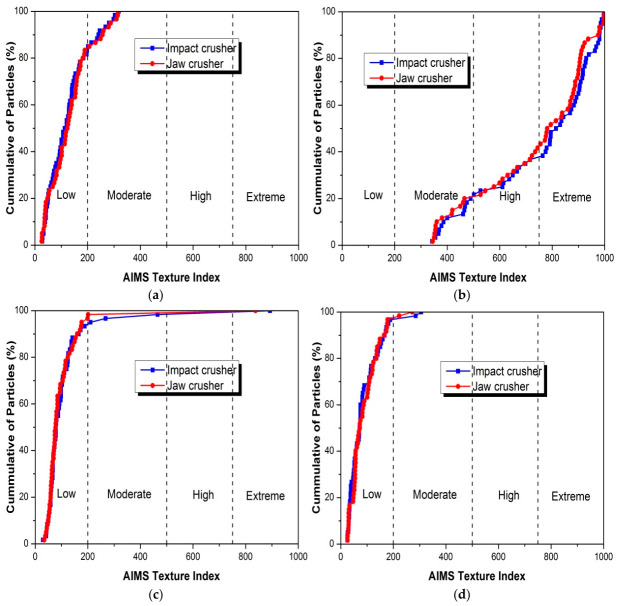

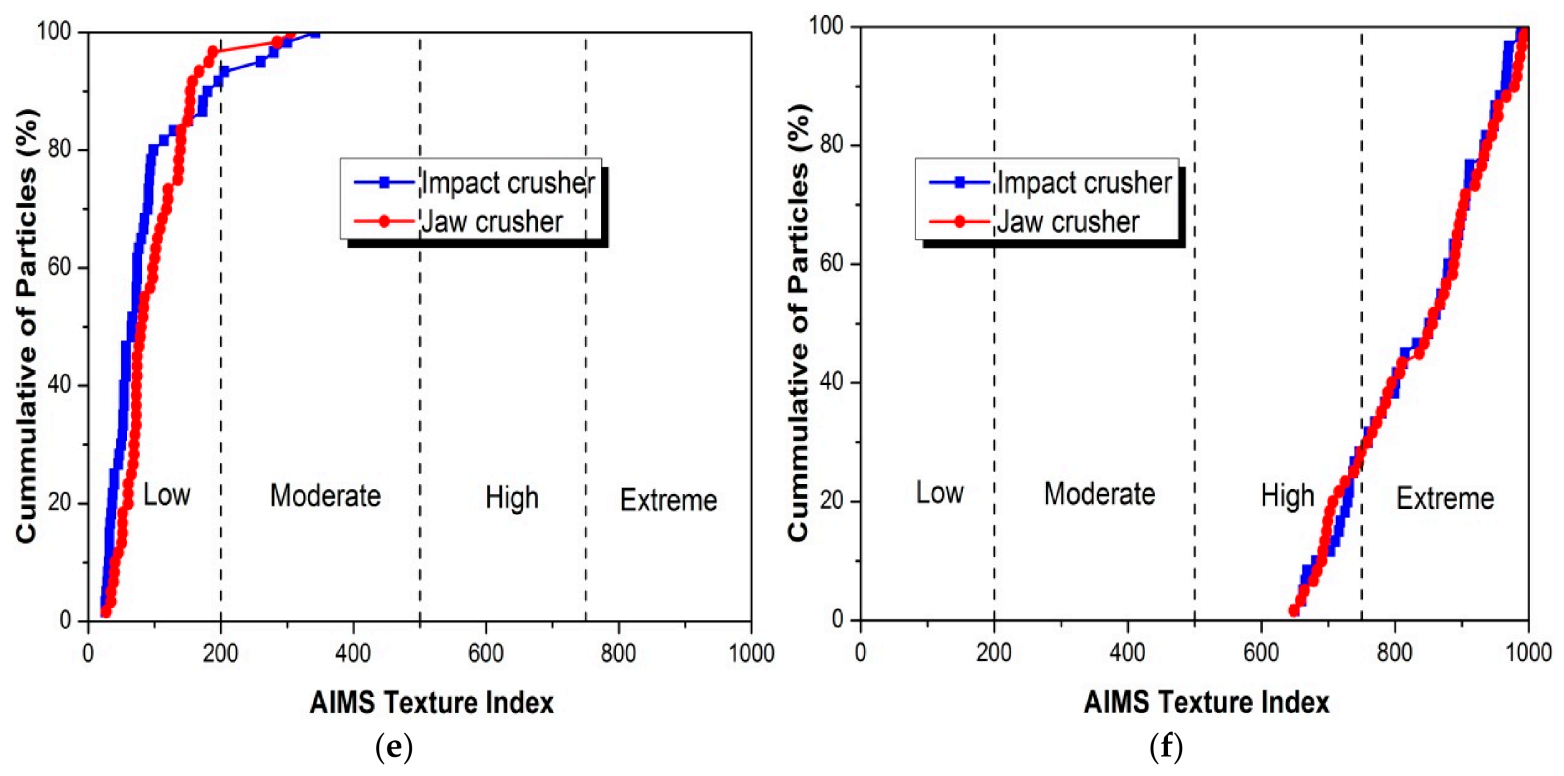
Texture comparison of aggregates under two crushing operations. (**a**) Quartz. (**b**) Amphibole. (**c**) Potash feldspar. (**d**) Sodium feldspar. (**e**) Calcite. (**f**) Pyroxene.

**Figure 9:**
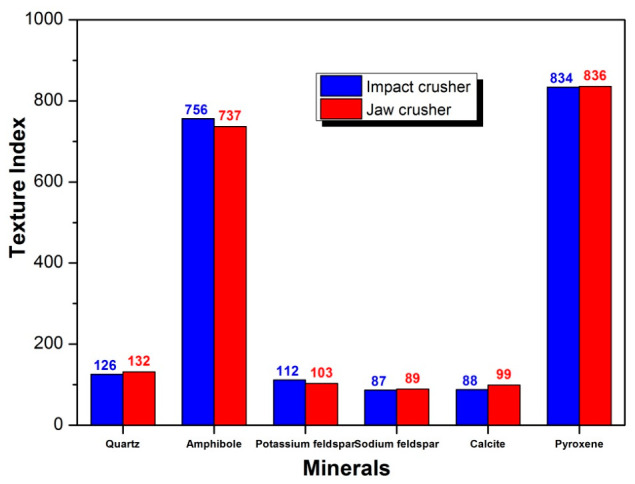
Mean texture index of different mineral aggregates.

Surface texture reflects the micro roughness of the aggregate surface, which influences the bonding strength between the aggregate and the asphalt mortar matrix. Figure 8 shows the plot of the surface texture for six mineral aggregates crushed by the jaw crusher and the impact crusher, respectively. Overall, there was no significant difference in the surface texture of the aggregates from these two crushers. However, the surface texture values of different mineral aggregates varied greatly. The quartz, potash feldspar, sodium feldspar and calcite had a higher percentage of aggregates in the low range of texture, while the amphibole and pyroxene had a higher percentage of particles in the high and extreme ranges of texture. In order to visually compare the texture differences of six mineral aggregates, the average texture was analyzed, as shown in Figure 9.

From Figure 9, it can be seen that the pyroxene and amphibole had much larger surface texture values, while the surface textures of the other four aggregates were much smaller. The 95% confidence interval of the average texture index for jaw-crushed aggregates (332.67) is close to that of impact-crushed aggregates (333.83), demonstrating no significant difference between the mean texture values of aggregates crushed under these two crushers and indicating that the surface texture of aggregates was mainly affected by their compositions and barely influenced by the crushing operations. So, the texture index of the aggregate might be controlled by its mineral crystal particle size. Therefore, the roughness of the aggregate surface can be improved by selecting aggregates containing more amphibole or pyroxene.

### 3.3. Sphericity

**Figure 10:**
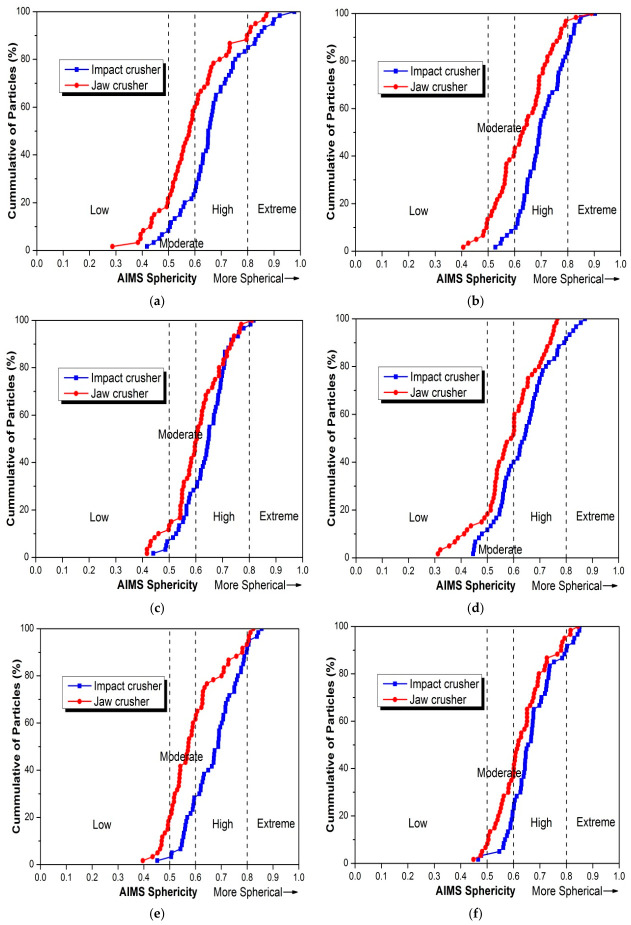
Sphericity comparison of aggregates under two crushing operations. (**a**) Quartz. (**b**) Amphibole. (**c**) Potash feldspar. (**d**) Sodium feldspar. (**e**) Calcite. (**f**) Pyroxene.

**Figure 11:**
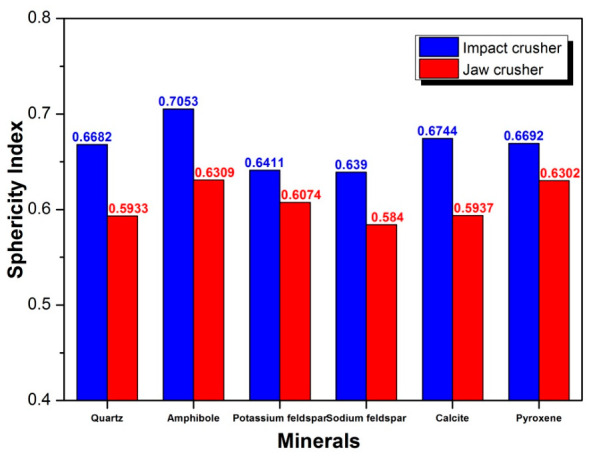
Mean sphericity index of different mineral aggregates.

Sphericity evaluates the overall roundness of particles. Figure 10a–f presents the cumulative percentage distribution of sphericity for six mineral aggregates crushed by jaw and impact crushers. It clearly demonstrates that aggregates from the impact crusher possess significantly higher sphericity values than those produced by the jaw crusher. This indicated that the aggregates produced by the impact crusher were more spherical. According to extensive existing research, aggregates with higher sphericity are more conducive to road stability. The mean values of sphericity for these six minerals are shown in Figure 11.

From Figure 11, it can be seen that the aggregates produced by the impact crusher had a higher mean sphericity compared to those by the jaw crusher. The 95% confidence interval of the average sphericity index for impact-crushed aggregates (0.666) is obviously higher than that of jaw-crushed aggregates (0.607), which is consistent with the above results. Thus, it can be concluded that the impact crusher produced more cubical aggregate particles compared to the jaw crusher.

### 3.4. F and E Ratio

**Figure 12:**
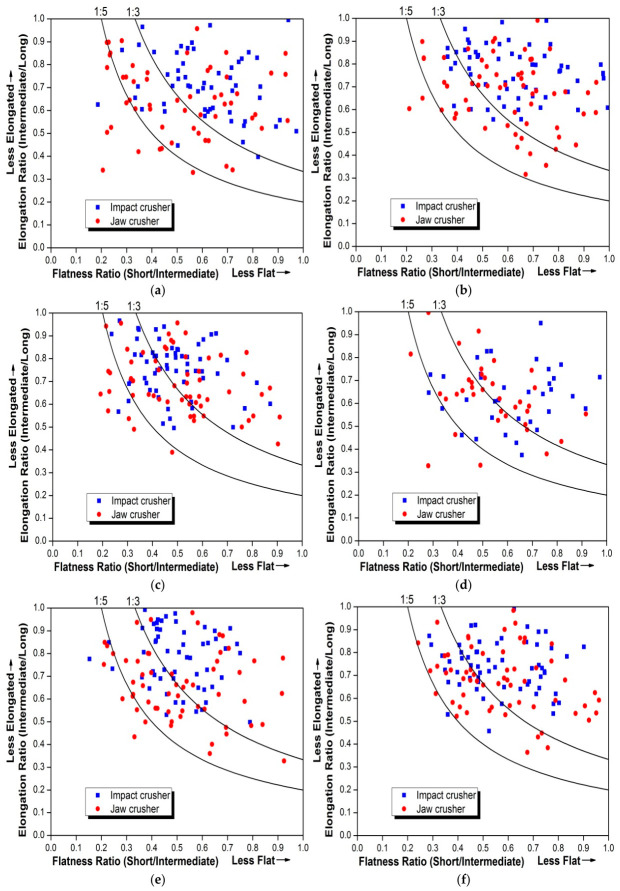
F and E ratio comparison of aggregates under two crushing operations. (**a**) Quartz. (**b**) Amphibole. (**c**) Potash feldspar. (**d**) Sodium feldspar. (**e**) Calcite. (**f**) Pyroxene.

**Figure 13:**
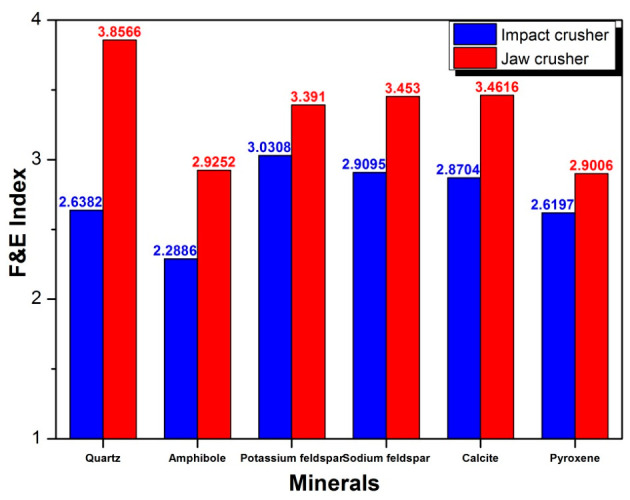
Mean F and E ratio of different mineral aggregates.

The F and E ratio reflects the content of flat and elongated particles within aggregates, which acts as a vital control index for raw materials used in asphalt pavement. A high proportion of flat–elongated aggregates will break the interlocking skeleton structure of the asphalt mixture and reduce the road-bearing capacity. The F and E ratio of six mineral aggregates crushed by the jaw crusher and the impact crusher are shown in Figure 12. It can be seen that the distribution of aggregates differed greatly for these two crushers. Obviously, the jaw crusher produced more aggregates with an aspect ratio of 1:3 and 1:5 (3 < F and E ratio < 5), while the impact crusher produced a higher percentage of particles with an aspect ratio of 1:3 (F and E ratio < 3). Since the coarse aggregates with excessively high F and E particles are prone to fracture under cyclic traffic loads on asphalt pavement, these aggregates are undesirable for road construction [12]. Therefore, aggregates crushed by a jaw crusher impair the pavement’s fatigue resistance under long-term vehicle cyclic loading. For a more intuitive comparison, the average values of F and E were drawn, as shown in Figure 13.

Since coarse aggregates with excessively high flat-and-elongated ratios are prone to fracture under cyclic traffic loads on asphalt pavement, these aggregates are undesirable for road construction. Therefore, aggregates crushed by a jaw crusher impair the pavement’s fatigue resistance under long-term vehicle cyclic loading.

From Figure 13, it can obviously be seen that all the mean F and E ratios of aggregates produced by the jaw crusher were bigger than those produced by the impact crusher. The 95% confidence interval of the average F and E ratio index for jaw-crushed aggregates (3.331) is markedly higher than that of impact-crushed aggregates (2.726). The jaw crusher produced more particles with an F and E of three or bigger than three, indicating that the aggregates crushed by the jaw crusher were more flat or elongated. In addition, the sorting of F and E of the six aggregates under the two crushers is significantly different, indicating that the F and E ratio of coarse aggregates was mainly affected by crushing operations. Moreover, the shape of quartz is most affected by crushers, while pyroxene is least affected. Therefore, based on the test results of this study, jaw crushers are not suggested for secondary crushing when processing aggregates with a high quartz content under the experimental conditions.

## 4. Conclusions

In this study, the effect of the mineral compositions and crushing operations on the morphologies of coarse aggregates was investigated. Six types of major rock-forming mineral aggregates were crushed using the jaw crusher and impact crusher, respectively. The morphology parameters of different aggregates produced by two crushers were measured and compared. Based on detailed results and discussion, the following conclusions can be drawn under the constraints of limited mineral samples and fixed crushing operating conditions.

(1) The angularity results showed that the jaw crusher can produce aggregates with higher angularity values compared to the impact crusher, especially for the calcite and pyroxene aggregates. The angularity difference between various minerals might be caused by the hardness of the mineral itself, and the angularity of the harder mineral is less affected by the crusher. Therefore, for aggregates containing a lot of calcite or pyroxene, the jaw crusher is preferentially adopted to effectively improve aggregate angularity.
(2) There was no significant difference in the surface texture of the aggregates from these two crushers, while the surface texture values of different mineral aggregates varied greatly, indicating that the surface texture of aggregates was mainly affected by their compositions and barely influenced by the crushing operations. Thus, for aggregates containing more amphibole or pyroxene, either crusher is available to guarantee excellent surface roughness of aggregates.
(3) The impact crusher produced more cubical aggregate particles compared to the jaw crusher, whereas the jaw crusher produced aggregates with more elongated or flat particles. So, the aggregates crushed by the jaw crusher were detrimental to the fatigue resistance of the pavement. Therefore, for aggregates containing lots of quartz, the impact crusher is prioritized to cut down flat and elongated particles and maintain good pavement fatigue resistance.

The crushing laws obtained from mineral aggregates in this study can provide intuitive references for crushing process selection in asphalt mixing plants and raw aggregate screening for pavements. With dozens of aggregate particles in each data group, the adequate sample size reduces statistical uncertainty and ensures reliable conclusions to support improvements in the long-term fatigue durability of asphalt mixtures during engineering construction. Future research will focus on the coupling effects of multiple factors, including aggregate origin, mixed mineral composition, and crushing parameters, on particle morphological characteristics.

## Figures and Tables

**Table 1:** Sources of major rock-forming minerals.

Minerals	Quartz	Amphibole	Potassium Feldspar	Sodium Feldspar	Calcite	Pyroxene
Source	Zhuji	Boshan	Qinhuangdao	Keketuohai	Jiande	Zhuji
Moh’s hardness	7	6.5	6	6	3	5

**Table 2:** Morphological parameters of coarse aggregate.

Morphology Parameters	Calculation Equations	Distribution
Angularity	$Angularity=\frac{1}{\frac{n}{3}-1}\sum_{i=1}^{n-3}\left|\theta_{i}-\theta_{i+3}\right|$ (*n* = number of points; *i* = *i*th point on the edge of particle; *θ* = orientation angle of edge points)	Low ≤ 2100 2100 < Moderate ≤ 3975 3975 < High ≤ 5400 5400 < Extreme ≤ 10,000
Texture	$Texture=\frac{1}{3N}\sum_{i=1}^{3}\sum_{j=1}^{N}{\left[D_{i,j}(x,y)\right]}^{2}$ (*N* = number of coefficients in an image; *i* = 1, 2 or 3 for detailed images; *j* = wavelet index; *D* = decomposition function)	Low ≤ 200 200 < Moderate ≤ 500 500 < High ≤ 750 750 < Extreme ≤ 1000
Sphericity	$Sphericity=\sqrt[{3}]{\frac{d_{S}d_{I}}{d_{L}^{2}}}$ (*d_L_* = longest dimension of the aggregate; *d_S_* = shortest dimension of the aggregate; *d_I_* = intermediate dimension of the aggregate)	Low ≤ 0.5 0.5 < Moderate ≤ 0.6 0.6 < High ≤ 0.8 0.8 < Extreme ≤ 1.0
F&E	$F\&E=\frac{d_{L}}{d_{S}}$ (meanings of *d_L_* and *d_S_* are the same as above)	-

Note: The classification thresholds for angularity, texture and sphericity are defined according to ref. [25].

## Data Availability

The original contributions presented in this study are included in the article. Further inquiries can be directed to the corresponding author.
